# Supplementary material for: CryoEM and computer simulations reveal a novel kinase conformational switch in bacterial chemotaxis signaling
Source: eLife. 2015 Nov 19;4:e08419. doi: 10.7554/eLife.08419 (PMC6746300; doi:10.7554/eLife.08419)
Supplement: Supplementary file 1. — Residues participating in a given interface but not associated with particular partners are listed separately for each domain. Residues that interact significantly (>50% of frames) are listed as a pair in a separate row. Interactions unique to this study are listed in green. Where ambiguous, residue pairs involving a receptor bound to CheA-P5, CheW from a CheA-P4/CheW ring or CheW from a CheW-only ring are denoted with a (1), (2) or (3) respectively. ** Signifies interfaces taken directly from experimental structures. Recent references pertaining to each protein-protein interface are given. DOI: http://dx.doi.org/10.7554/eLife.08419.023 [file elife-08419-supp1.docx]

| Interface | Domain | Residues | References |
| --- | --- | --- | --- |
| CheA-P5/Receptor | CheA-P5 | L547, I560, I563, I566, L629 | 23, 24, 25, 27, 28 |
|  | Receptor | L362, L365, N366, A368, I369, A372 |  |
|  | CheA-P5/Receptor | D564/R373(1) |  |
| CheW/Receptor | CheW | L14, V27, I30, V33, V98, V101 | 23, 24, 25, 26, Present study |
|  | Receptor | L362, L365, N366, A368, I369, A372 |  |
|  | CheW/Receptor | K9/R376(1&3), E10/R379(1&3), E12/R379(2), D28/R373(2), E31/R373(2) |  |
| CheA-P5/CheW interface I ** | CheA-P5 (subdomain 1) | L554, L599, I601, L640, V643, F644, V647, F650, A653, I655, I661, L663 | 20, 23, 24, 29, 30 |
|  | CheW (subdomain 2) | T40, P41, V42, P43, V49, V52,  I59, P61, V63, V89, V91, I145 |  |
|  | CheA-P5/CheW | K642/D88, E646/R46, E649/K44, |  |
| CheA-P5/CheW interface II ** | CheA-P5 (subdomain 2) | Q575, V577, Q578, V582, V584, V589,  P591 | 23, 24 |
|  | CheW (subdomain 1) | T112, N113, V114, S115, F118, L125,  L132, I134 |  |
|  | CheA-P5/CheW | R580/D116, E588/K127 |  |
| CheW/CheW | CheW (subdomain 1) | Q21, L69, I71, T112, N113, V114,  S115, F118, L125, L132, I134 | Present study. |
|  | CheW (subdomain 2) | T40, P41, V42, P43, V49, V52,  I59, P61, V63, V89, V91, I145 |  |
|  | CheW/CheW | D116/R46 |  |
| CheA-P3/Receptor (Non-dipped) | CheA-P3/Receptor | D333/K390(2), D345/R379(2) | 25, Present study. |
| CheA-P3/CheA-P4 (Non-dipped) | CheA-P3/CheA-P4 | K352/E390, R354/D392, D304/R393 | 19, Present study. |
| CheA-P4/Receptor (Dipped) | CheA-P4/Receptor | E390/R379(2) | Present study. |
| CheA-P3/CheA-P4 (Dipped) | CheA-P3/CheA-P4 | K352/E390, R354/D392, R297/E397 | 19, Present study. |
| Receptor/Receptor | homodimer/homodimer | E370-R373, E351-R403, E387-R389 | 18, Present study. |

Supplementary File 1
